# Association of white matter hyperintensities with lipoprotein (a) levels: insights from a cohort study

**DOI:** 10.3389/fneur.2024.1476005

**Published:** 2024-12-06

**Authors:** Fawaz F. Alotaibi, Gamal Mohamed, Sawsan S. Bakry, Mohammed Alqahtani, Hussain BinAmir, Ammar AlKawi, Abdulrahman A. Alreshaid, Mohamed AlZawahmaha, Adel Alhazzani, Ashfaq Shuaib, Fahad S. Al-Ajlan

**Affiliations:** ^1^Neuroscience Center, King Faisal Specialist Hospital and Research Center, Riyadh, Saudi Arabia; ^2^Department of Biostatistics, Epidemiology, and Scientific Computing, King Faisal Specialist Hospital and Research Centre, Riyadh, Saudi Arabia; ^3^Neuroscience Center, King Faisal Specialist Hospital, Alfaisal University, Riyadh, Saudi Arabia; ^4^Department of Medicine, University of Alberta, Edmonton, AB, Canada

**Keywords:** cerebrovascular disease, stroke, white matter disease, genetics, lipoprotein (a), C-reactive protein

## Abstract

**Background:**

Little is known about the relationship between lipoprotein (a) [Lp(a)] and cerebral white matter hyperintensities (WMH). The aim of the study was to examine if elevated Lp(a) levels are associated with higher burden of WMH.

**Methods:**

We retrospectively investigated associations between Lp(a) and the burden of WMH among patients with confirmed diagnosis of acute ischemic stroke or transient ischemic attacks. WMH burden was assessed using 3-Tesla brain MRI and graded according to the Fazekas score. Multivariable models were generated to determine the contribution of Lp(a) to the presence and extent of WMH.

**Results:**

One hundred and fifty-three patients were included (mean age, 45.9 years; 35.9% women). When the study population was stratified by Lp(a) level into three categories, low (<75 nmol/L), moderate (75 to <125 nmol/L), and high (≥125 nmol/L), the distribution of the three groups was 60.8, 15.0 and 24.2%, respectively. High Lp(a) Level was associated with higher burden of both periventricular WMH and deep WMH compared to the lower level (odds ratio [OR], 4.4; 95% confidence interval [CI], 1.60–12.07; *p* = 0.004; and OR, 5.6; CI, 1.69–14.7; *p* = 0.001, respectively).

**Conclusion:**

We show in this cohort of patients that a higher burden of WMH was observed in patients with higher level of Lp(a). Further studies are needed to confirm this observation and assess whether lowering Lp(a) level may be a potential therapeutic target for mitigating the development of WMH.

## Introduction

Lipoprotein a [Lp(a)] is a low-density lipoprotein (LDL) particle (1), consisting of apolipoprotein B100, and glycoprotein apolipoprotein (a) (2) and has emerged as an independent risk factor for atherosclerotic cardiovascular disease (3). Proposed mechanisms include its role in foam cell formation, promotion of cholesterol deposition and increased immunological responses (4).

White matter hyperintensities (WMH) of presumed vascular origin increases with age, hypertension, diabetes and other vascular risk factors (5) and is associated with an increased risk of dementia, stroke, worse post-stroke outcomes and higher mortality. There is limited data regarding the association between Lp(a), WMH, and stroke.

In this study, we report the association between Lp(a) and WMH in patients with acute ischemic stroke or transient ischemic attacks (TIAs).

## Methods

### Study population

This study was approved by the institutional review board of King Faisal Specialist Hospital and Research Center in Riyadh, Saudi Arabia, number (DNS/952/45). A retrospective analysis was conducted on data obtained from 153 patients admitted to the comprehensive neurovascular unit between January 2023 and March 2024, following a diagnosis of acute ischemic stroke or transient ischemic attacks. LP(a) and high sensitivity C-reactive protein (hsCRP) were measured during their hospital admission. The Lp(a) levels were evaluated as low (<75 nmol/L), moderate (75 to <125 nmol/L), and high (≥125 nmol/L).

### MRI acquisition and assessment

Brain MRI scans were acquired using a 3-Tesla MRI scanner (insert scanner model and manufacturer) with a standard head coil. The imaging protocol included T1-weighted, T2-weighted, DWI and FLAIR with slice thickness of 5 mm. MRI images were independently reviewed by two stroke neurologists blinded to the clinical information. Periventricular WMH (PVWMH) and deep WMH (DWMH) burden were assessed separately on FLAIR and T2WI sequences and were graded according to the Fazekas scale into none, mild, moderate, and severe (6).

### Statistical analysis

Quantitative variables were summarized as mean (SD) and median (interquartile range) and were compared with one-way ANOVA, whereas qualitative variables were summarized as frequencies and percentages and were compared with Chi square. The effects of the independent variables on Fazekas were investigated using univariate and stepwise multivariable logistic regression. *p*-value <0.05 was considered significant. Relationships among the different Lp(a) and hsCRP were determined using the Pearson product moment correlation coefficient (r). Stata 18 was used for the analysis.

## Results

One hundred and fifty-three patients were included (mean age, 45.9 years; 35.9% women). There was no significant difference in the demographics as shown in Table 1. The etiology of stroke was distributed as follows, large artery atherosclerosis accounted for 35%, small vessel disease comprised 34%, cardioembolic strokes were 9.8%, strokes of other determined etiology were 6.5%, and strokes of undetermined etiology made up 15%. The mean NIHSS score was 9. Patients stratified by Lp(a) into three categories, low (<75 nmol/L), moderate (75 to <125 nmol/L) and high (≥125 nmol/L). The distribution of Lp(a) in the three groups was 60.8, 15.0 and 24.2%, respectively. The hsCRP levels and Fazekas scores (periventricular and deep) were significantly higher with increased Lp(a) (*p* = 0.015. and *p* = <0.001 respectively).

**Table 1:** Baseline characteristics of the study cohort stratified according to the lipoprotein a levels.

Variables	Lipoprotein (a) stratification				*p*-value
	Low (<75 nmol/L)	Moderate (75 to < 125 nmol/L)	Severe (≥125 nmol/L)	Total	
	N % 93 (60.8%)	N % 23 (15.0%)	N % 37 (24.2%)	N % 153 (100.0)	
Male sex, n (%)	61 (65.6%)	15 (65.2%)	22 (59.5%)	98 (64.1%)	0.799
Diabetes, n (%)	39 (41.9%)	10 (43.5%)	15 (40.5%)	64 (41.8%)	0.975
Hypertension, n (%)	37 (39.8%)	9 (39.1%)	21 (56.8%)	67 (43.8%)	0.189
Dyslipidemia, n (%)	31 (33.3%)	10 (43.5%)	17 (44.7%)	58 (37.7%)	0.342
Smoking, n (%)	21 (22.6%)	4 (17.4%)	6 (15.8%)	31 (20.1%)	0.670
Atrial fibrillation, n (%)	6 (6.5%)	0 (0.0%)	0 (0.0%)	6 (3.9%)	0.133
Any Cardiac disease, n (%)	14 (15.2%)	5 (21.7%)	10 (27.0%)	29 (19.1%)	0.285
Recurrent ischemic stroke, n (%)	24 (25.8%)	8 (34.8)8	9 (23.7%)	41 (26.6%)	0.635
Family history of stroke, n (%)	2 (2.2%)	1 (4.3%)	1 (2.6%)	4 (2.6%)	0.839
hsCRP ≥ 2, n (%)	1 (1.1%)	3 (13.0%)	4 (10.8%)	8 (5.2%)	0.015
Periventricular Fazekas score (none/mild), n (%)	76 (81.7%)	15 (65.2%)	18 (47.4%)	109 (70.8%)	<0.001
Periventricular Fazekas score (moderate/severe), n (%)	17 (18.3%)	8 (34.8%)	19 (51.4%)	44 (28.8%)	
Deep Fazekas score (none/mild), n (%)	74 (79.6%)	14 (60.9%)	17 (44.7%)	105 (68.2%)	<0.001
Deep Fazekas score (moderate/severe), n (%)	19 (20.4%)	9 (39.1%)	20 (54.1%)	48 (31.4%)	

**Figure 1:**
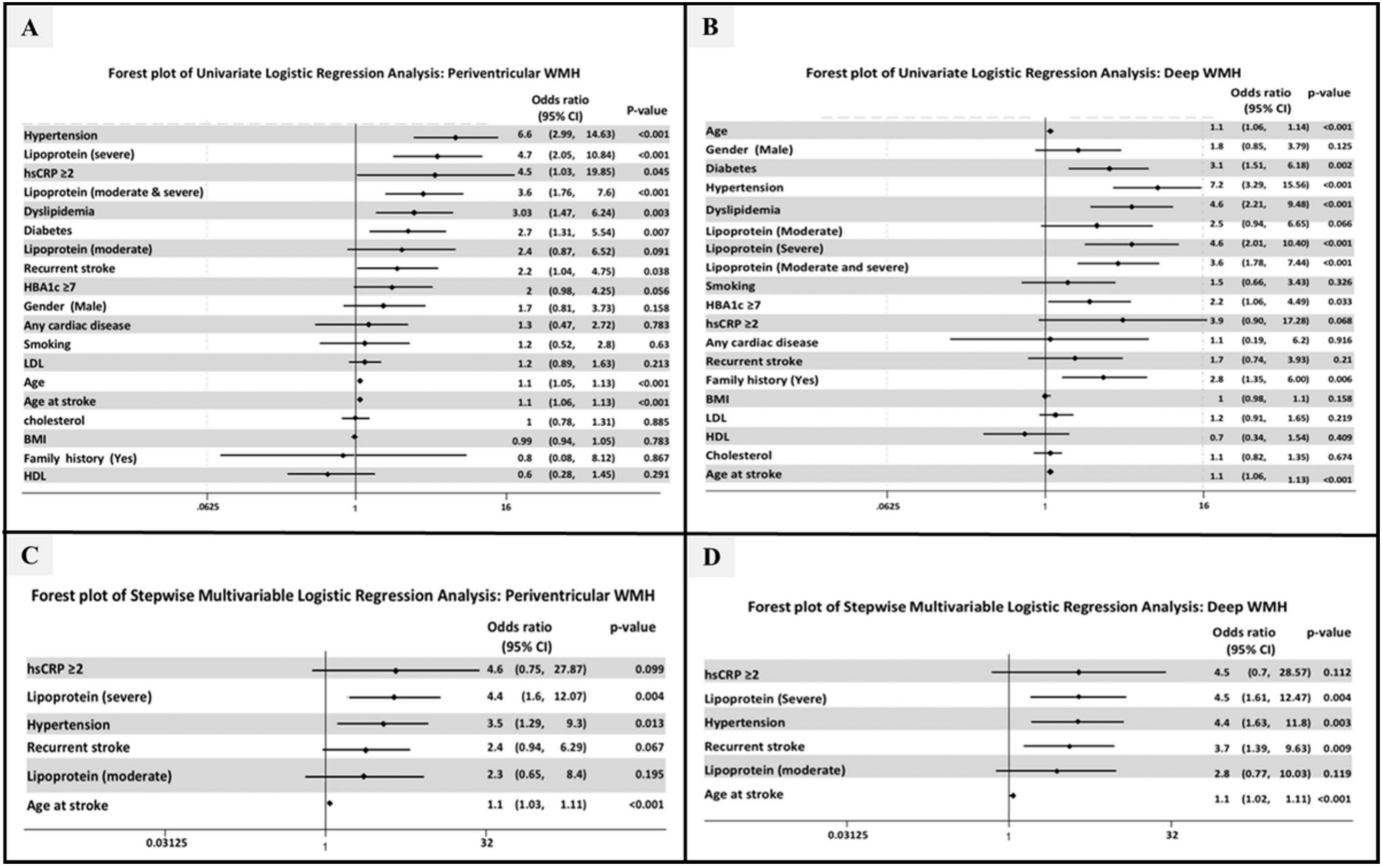
Forest plots showing the results of univariate and multivariable logistic regression analysis for WMH burden. **(A,B)** Forest plots for univariate analysis for periventricular WMH and deep WMH, respectively. **(C,D)** Forest plots for multivariate analysis for periventricular WMH and deep WMH, respectively.

Univariate logistic regression analysis revealed that high Lp(a) level was significantly associated with higher score of PVWMH (OR = 4.7, 95% CI:2.05–10.84, *p* < 0.001) and DWMH (OR = 4.6, 95% CI:2.01–10.40, *p* = <0.001), hsCRP level ≥ 2 was associated with higher Fazekas scores (OR = 4.5, 95% CI: 1.03–19.85, *p* < 0.045) (Figures 1A,B).

**Figure 2:**
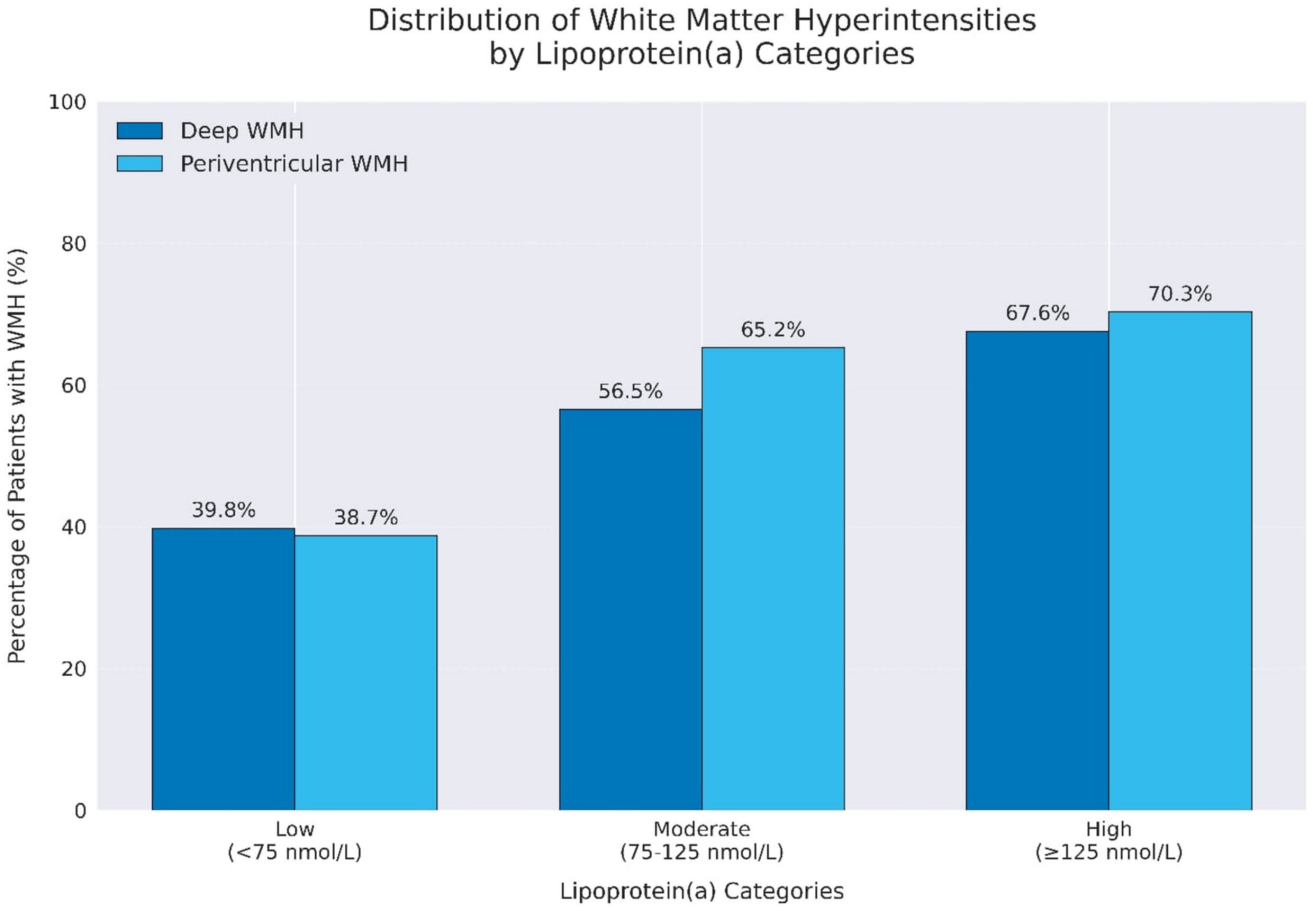
The distribution of white mater hyperintensities by lipoprotein a categories.

Stepwise multivariable logistic regression analysis showed that high Lp(a) remained a significant predictor of higher Fazekas scores for both PVWMH (OR = 4.4, 95% CI: 1.6–12.07, *p* = 0.004) and DWMH (OR = 5.4, 95% CI: 1.96–14.70, *p* = 0.001) (Figures 1C,D). The odds ratio for moderate and high Fazekas score for PVWMH and DWMH are illustrated in Forest plots (Figures 1A,B, 2). There was an increase in the percentage of WMH with moderate and high scores as Lp(a), increasing from 18.3% in the low group to 52.6% in the high group for PVWMH scores and from 20.4 to 55.3% for DWMT scores (Figure 2). We observed low correlations between the Lp(a) level and hsCRP (*r* = 0.369) (Supplementary Figure S1).

## Discussion

Numerous studies have investigated the relationship between high Lp(a) and cardiovascular disease and stroke (7). Lp(a) is a risk factor for stroke (8) and is associated with an increased risk of recurrent vascular events in patients with acute stroke (9). Our study adds two important observations between Lp(a) and the burden of WMH. Firstly, we found a positive association between increasing Lp(a) levels and higher burden of WMH. After excluding the six patients with atrial fibrillation from the low LP(a) group, the analysis still shows a statistically significant association between LP(a) levels and the presence of WMH (*p* = 0.0108). Secondly, similar to reports in cardiac diseases, we observed an association between the impact of Lp(a) and the hsCRP level.

The potential mechanisms underlying the association between Lp(a) and cerebral WMH are likely multifactorial, including enhanced inflammation, accelerated atherosclerotic plaque formation and embolization, leading to cerebral microvascular occlusion and ischemic injury (10). The increase in WMH with higher Lp(a) observed in our study suggests that microvascular damage precedes the onset of symptomatic stroke. The higher levels of hsCRP observed in the patients with higher Lp(a) might point to inflammation contributing to the WMH.

Limitation of the study is that recruiting patients in the acute stroke phase may introduce selection bias, as it excludes patients with different types of strokes who do not present to the hospital under a similar setting. Further limitation is that this is a cohort study, and we cannot draw conclusions regarding causality. The relationship between higher Lp(a) and high hsCRP is intriguing. However, our sample size was small and requires confirmation from prospective studies whether this may have a synergistic impact on WMH.

In conclusion, to our knowledge, this is the first stroke cohort study using 3-Tesla MRI to systematically assess the burden of periventricular and deep WMH stratified by Lp(a) levels. We show a higher burden of WMH in patients with higher level of Lp(a). Further studies are needed to confirm this observation and assess whether lowering Lp(a) level may be a potential therapeutic target for mitigating the development of WMH.

## Data Availability

The original contributions presented in the study are included in the article/Supplementary material, further inquiries can be directed to the corresponding author/s.
