# Supplementary figures and images for: Association of white matter hyperintensities with lipoprotein (a) levels: insights from a cohort study
Source: Front Neurol. 2024 Dec 6;15:1476005. doi: 10.3389/fneur.2024.1476005 (PMC11663010; doi:10.3389/fneur.2024.1476005)

**Supplement Figure 1:** Correlation between lipoprotein (a) and hsCRP.

**| lipopr~a CRP**

**-------------+------------------**

**lipoprteina | 1.0000**

**CRP | 0.3690 1.0000**


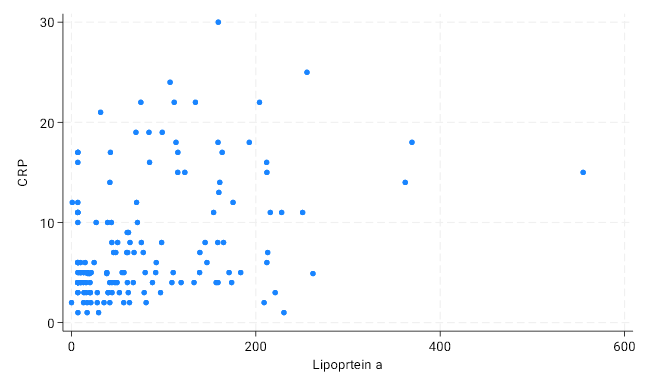

Supplement: Supplementary file 1 [file Data_Sheet_1.DOCX]
